# PiHelper: an open source framework for drug-target and antibody-target data

**DOI:** 10.1093/bioinformatics/btt345

**Published:** 2013-07-08

**Authors:** Bülent Arman Aksoy, Jianjiong Gao, Gideon Dresdner, Weiqing Wang, Alex Root, Xiaohong Jing, Ethan Cerami, Chris Sander

**Affiliations:** ^1^Computational Biology Center, Memorial Sloan-Kettering Cancer Center, NY 10065, ^2^Tri-Institutional Training Program in Computational Biology and Medicine, NY 10065 and ^3^Blueprint Medicines, Cambridge, MA 02142, USA

## Abstract

**Motivation:** The interaction between drugs and their targets, often proteins, and between antibodies and their targets, is important for planning and analyzing investigational and therapeutic interventions in many biological systems. Although drug-target and antibody-target datasets are available in separate databases, they are not publicly available in an integrated bioinformatics resource. As medical therapeutics, especially in cancer, increasingly uses targeted drugs and measures their effects on biomolecular profiles, there is an unmet need for a user-friendly toolset that allows researchers to comprehensively and conveniently access and query information about drugs, antibodies and their targets.

**Summary:** The PiHelper framework integrates human drug-target and antibody-target associations from publicly available resources to help meet the needs of researchers in systems pharmacology, perturbation biology and proteomics. PiHelper has utilities to (i) import drug- and antibody-target information; (ii) search the associations either programmatically or through a web user interface (UI); (iii) visualize the data interactively in a network; and (iv) export relationships for use in publications or other analysis tools.

**Availability:** PiHelper is a free software under the GNU Lesser General Public License (LGPL) v3.0. Source code and documentation are at http://bit.ly/pihelper. We plan to coordinate contributions from the community by managing future releases.

**Contact:** pihelper@cbio.mskcc.org

## 1 INTRODUCTION

In cancer biology, systems pharmacology and perturbation biology, researchers designing targeted drug experiments often need to choose targeted drugs and antibodies of interest for their experimental studies. For such studies, drug- and antibody-target databases are valuable resources and are increasingly publicly available in computable formats. Unfortunately, this information is in separate databases that use mostly incompatible formats, making it difficult to integrate data across different resources. This, coupled with strict constraints on distribution of the data, hinders access to up-to-date integrated data.

Here we describe an open-source framework, PiHelper, for easy aggregation, integration and visualization of drug- and antibody-target data from multiple sources. PiHelper provides a platform-independent command-line tool to help users, with minimal configuration, import and export drug- and antibody-target information in a human- and gene-centric manner; a Java application programming interface and a REST-ful (Representational State Transfer) web service to facilitate programmatic access to the aggregated data; and a web-based UI to help users query data in a gene-centric manner and export the results as an image or undirected binary network.

We believe PiHelper will facilitate hypothesis generation and design of new experiments by enabling researchers to access and query integrated drug-target and antibody-target data from multiple resources in an automatic way.

## 2 COMPONENTS

### 2.1 Administration module

The administration module provides a command-line interface for users to import data into a database or export the aggregated data to tab-delimited format for further analysis. The *importer* component supports automatic fetching of background gene information, gene sets, gene-centric drug-target and antibody-target annotations from multiple resources (Table 1). Importing data from these resources is accomplished in an automatic manner through PiHelper’s admin command-line interface. The admin module contains specific data converters for each resource and frees the user from handling different file formats and merging data across resources. The user also has the option to import drug- and antibody-target data from custom tab-delimited files.

**Table 1.** PiHelper enables integration of 10 publicly available drug-target and drug-antibody resources

Data resource	Type of data
DrugBank (Knox *et al.*, 2011)	Drug-target
KEGG Drug (Kanehisa *et al.*, 2012)	Drug-target
Rask-Andersen *et al.*, 2011	Drug-target
GDSC (Yang *et al.*, 2013)	Drug-target
Garnett *et al.*, 2012	Drug-target
Cancer.gov http://cancer.gov	Drug-annotation
The Human Protein Atlas (Uhlen *et al.*, 2010)	Antibody-target
Tibes *et al.*, 2006	Antibody-target
Pawlak *et al.*, 2002	Antibody-target
Pathway Commons (Cerami *et al.*, 2011)	Gene-sets

*Note*: KEGG, Kyoto Encyclopedia of Genes and Genomes; GDSC, The Genomics of Drug Sensitivity in Cancer

Once the database is populated through the admin tool, the *exporter* component can be used to export all drug and antibody data to a tab-delimited text format (TSV). These files can then be used for further analysis tools, *e.g.* by importing the data into Cytoscape as a binary network and running graph-based queries or visualizing larger networks (Shannon *et al.*, 2003).

### 2.2 Web-based UI

**Fig. 1.**
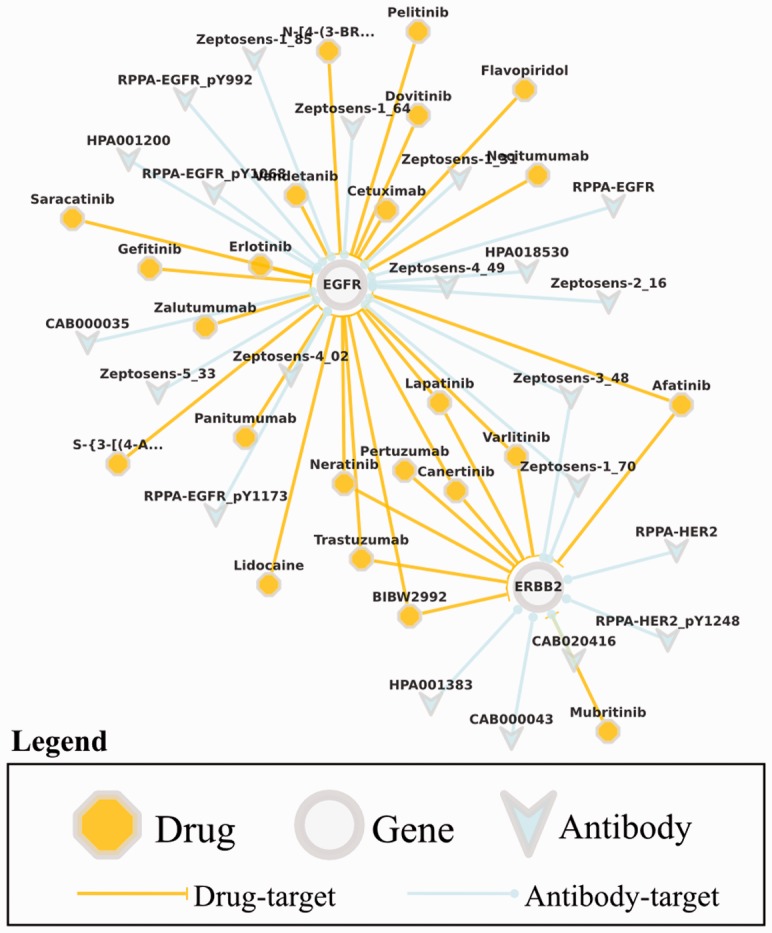
Sample target network visualization: results showing all drug and antibody associations of *EGFR* and *ERBB2* genes. PiHelper web UI allows exporting the network to various formats, e.g. SVG and Simple Interaction Formats

The web-based UI distributed as a part of PiHelper enables users to query antibodies and drugs in a gene-centric manner (Povey *et al.*, 2001). It also helps visualize the results as a binary network and export the final network in either scalable vector graphics (SVG), portable network graphics, GraphML or simple interaction formats. The visualization of the query results as an interactive network is accomplished through the Cytoscape Web library (Lopes *et al.*, 2010). The web-based UI features automatic validation of the gene names, pre-loaded gene sets representing most of the well-known canonical pathways in the query page; details for a gene, drug, antibody or the targeting interaction upon clicking on the corresponding element within the network; and options to expand the network based on either genes or drugs in the network and to download the network for external use (Fig. 1).

### 2.3 Core module

The core module provides the model Java classes and basic finder methods. The model classes consist of basic elements, such as Drug, Gene and DrugTarget, which capture drug– and antibody–gene relationships. These elements, together with their querying methods, help developers build custom applications or analysis tools that depend on drug or antibody annotation data.

Besides the Java application programming interface, the core module also includes a web service component that provides basic querying methods through REST protocols. The web service supports obtaining the results in either JavaScript Object Notation or HyperText Markup Language. The former provides flexibility for developers who prefer other programming languages than Java; the latter enables users to interact with the database via their web browser of choice.
